# Gender Differences in the Relationships Between Coach Transformational Leadership and Player Satisfaction and Commitment: A Meta-Analytic Review

**DOI:** 10.3389/fpsyg.2022.915391

**Published:** 2022-06-21

**Authors:** Hyun-Duck Kim, Angelita Bautista Cruz

**Affiliations:** ^1^Department of Sport Marketing, Keimyung University, Daegu, South Korea; ^2^Department of Physical Education, Keimyung University, Daegu, South Korea

**Keywords:** MLQ, exercise commitment, athletic satisfaction, sustainable coach leadership, coach-athlete interaction, gender dyad

## Abstract

This study meta-analyzed the relationships between coach transformational leadership and player satisfaction and commitment. We also examined the potential moderating effect of player gender on these relationships. In total, 182 effect sizes were obtained from 26 studies comprised of 6,715 participants. The analyses revealed that the overall direct effect of transformational leadership was moderate on both athletic satisfaction and exercise commitment. The effect of charismatic construct of transformational leadership was moderate on athletic satisfaction as well as exercise commitment. Finally, player gender was found to moderate the effects of the relationship between transformational leadership and athletic satisfaction and exercise commitment of players. Specifically, female players' satisfaction and commitment were more positively affected by transformational leadership compared with their male counterparts. Our findings suggests that effective leadership in sports is dependent on the interaction among leadership behaviors of the coach, personal characteristics of the players, and situational factors and highlights the importance of transformational leadership as an important requirement for creating a more positive and sustainable sports environment.

## Introduction

Studies on sports leadership have been ongoing for the past several decades because the complexities and multifaceted components of sports can have profound implications on the athletic participation of players or teams (Gilbert and Trudel, 2004; Weinberg and Gould, 2015; Kim and Cruz, 2016; Turnnidge and Côté, 2018; Sheehy et al., 2019; Evans and Pfister, 2021; Michalski and Lee, 2021). In a sports environment, coaches are sports leaders with diverse functions for achieving the desired goals, objectives, and performance pursuits of a player or team (Weinberg and Gould, 2015). These functions are not restricted to imparting technical and tactical knowledge, but also extend to offering support, motivation, and encouragement to players, advocating high performance expectations, and displaying the integrity and proper conduct fitting of a coach (Morton et al., 2014). This behavior, when perceived by players, is believed to affect their sport-related cognition and performance (Price and Weiss, 2013; Stenling and Tafvelin, 2014; Bormann et al., 2016; Kim and Cruz, 2016; Cheval et al., 2017; Ekstrand et al., 2018). It is therefore imperative to understand how leadership behaviors displayed by sports coaches relate to player cognitive and behavioral responses in order to identify which coaching behaviors promote or undermine sport-related outcomes in players. Identifying the degree on how leadership behaviors of coaches affect players' sport-related outcomes can facilitate the creation of appropriate intervention programs related to coach leadership development.

Sports coaches are generally recognized as leaders in sports that can spearhead players to reach their maximum potential in achieving sports success. In contrast, coaches as a leader can also be a potential factor influencing players' poor performance. Accordingly, researchers in the area of sports leadership are continuously investigating the process of how leadership of coaches influence sport-related outcomes of players. Leadership in sports is commonly explained with the Chelladurai's multidimensional model of leadership (MML) (1990). The MML is a leadership framework specifically developed for sports and physical activity, which suggests that the satisfaction and performance of players are dependent on coaches' leadership behaviors, and these behaviors are determined by three variables: the situation, leader, and member (Chelladurai, 1990). To measure leadership behaviors following this framework, the Leadership Scale for Sport (LSS) (Chelladurai and Saleh, 1980) was developed to evaluate a coach's training and instruction, decision-making style, and motivational behaviors in a sporting environment. Based on the results of leadership studies following this perspective, satisfaction and performance were indeed found to be associated with coach leadership behavior (Aoyagi et al., 2008; Prati and Pietrantoni, 2013; Kao et al., 2015; Kim and Cruz, 2016). Other consequences of leadership have also been found to be affected by leadership behaviors, such as cohesion (Kim and Cruz, 2016; Nascimento-Junior et al., 2018), motivation (Amorose and Horn, 2000; DoYoung et al., 2010), and commitment (Im et al., 2012; Berestetska, 2016). The results from these studies provide evidence supporting the MML framework in explaining effective leadership in a sporting context, particularly in understanding the leadership styles and behaviors of coaches, which affect player athletic participation.

Recently, the transformational leadership perspective has been applied to the sports context by researchers to understand leader effectiveness in sports. Transformational leadership is a leadership perspective adopted from organizational psychology (Bass, 1985, 1990, 1997; Bass and Riggio, 2006) that posits that the impact of leaders on follower motivation, morality, and performance is based on how the leader demonstrates transformational leadership. Transformational leadership has four interrelated components: (1) idealized influence, also referred to as charisma, which describes the leader's positive charisma that fosters trust and confidence in followers and leader's values that emphasize commitment to the organization's goals, (2) inspirational motivation, which refers to a leader's capacity to inspire followers to achieve their fullest potential and to properly communicate the importance of each follower's role in the successful operation of organizational goals, (3) intellectual stimulation, which refers to a leader's ability to encourage creativity and develop independent thinking in followers, and (4) individualized consideration, which relates to a leader's rapport and compassion for follower needs and concerns (Bass, 1997; Antonakis et al., 2003; Rowold, 2006). Moreover, this leadership perspective provides additional contribution to the influence of transactional leadership, which describes a leader's use of positive reinforcement (e.g., rewards or praise) when a follower performs desirable or positive behaviors and a leader's observant behaviors in recognizing any deviances from the set rules and regulations and taking immediate actions to correct them (Bass, 1997). The application of this leadership perspective in the sporting context was first introduced by Zacharatos et al. (2000), who were later followed by other researchers (Jang and Kim, 2008; Kim and Won, 2012; Choi, 2017; Kim et al., 2018; Sun and Lee, 2019).

In order to understand the concepts explaining transformational leadership, a measurement tool was developed called the Multifactor Leadership Questionnaire (MLQ-5X) (Bass and Avolio, 2000). The MLQ-5X is a widely used instrument to assess transformational leadership behaviors (Lowe et al., 1996; Rowold, 2006; Price and Weiss, 2013; Batista-Foguet et al., 2021; Kao et al., 2021; Malloy and Kavussanu, 2021). This instrument has nine factors: five on transformational leadership behavior, three on transactional leadership behavior, and one on laissez-faire leadership behavior.

The five transformational leadership factors are idealized influence-attributed, idealized influence-behavior, inspirational motivation, intellectual stimulation, and individualized consideration. Furthermore, the idealized influence and inspirational motivation factors of transformational leadership share similar concepts and are identified as charismatic dimensions of transformational leadership.

The three factors of transactional leadership are as follows: (1) contingent rewards, (2) active management by exception, and (3) passive management by exception. Contingent reward pertains to a leader's use of positive reinforcement (e.g., rewards or praise) when a follower performs desirable or positive behaviors. Active management by exception describes a leader's observant behaviors in recognizing any deviances from the set rules and regulations and taking immediate actions to correct them, whereas passive management by exception denotes the leader's actions to rectify mistakes only after the errors have been detected. Finally, laissez-faire is the most passive leadership approach, and has even been described as the absence of leadership (Antonakis et al., 2003; Rowold, 2006).

This MLQ instrument has been adopted in various sports setting to assess transformational leadership of coaches worldwide and findings showed that the psychological states of players can be affected by their coach's transformational leadership qualities (Cho and Ha, 2009; Eun, 2009; Hur, 2010; Lee et al., 2011; Seo, 2012; Choi et al., 2013; Lee and Yeo, 2013; Bum and Shin, 2015; Ryu and Park, 2021). In Korea for instance, charismatic and exceptional management behaviors among archery coaches significantly affect player commitment to exercise (Ryu and Park, 2021). Jang et al. (2011) reported that the transformational leadership of coaches has a significant effect on taekwondo player commitment. Interestingly, they also found that player exercise commitment was substantially affected by their coach's transactional leadership behavior. Cho and Ha (2009) found that both idealized influence-attributes and behavior and inspirational motivation transformational leadership of coaches were positively associated with sport commitment in tennis players.

Aside from commitment, player's satisfaction was also found to be affected by transformational leadership of coaches. Kim et al. (2018) reported that the transformational leadership constructs individual consideration, inspirational motivation, and idealized influence were associated with the sports satisfaction of taekwondo athletes. Kim and Won (2012) demonstrated that individual consideration, idealized influence, and intellectual stimulation significantly predicted player satisfaction. Hur (2010) found that not only coaches' inspirational motivation but also their idealized influences in building collective identity (behavior), trust, and confidence (attributed) significantly predicted satisfaction among skiers. The results of these studies provide evidence not only of the positive association but also the mixed results of transformational leadership with sport-related psychological variables of satisfaction and commitment. Sports satisfaction describes a sport participant's positive emotional state based on the assessment of the structures, processes, and outcomes related to one's athletic experiences (Chelladurai and Riemer, 1997). Sports commitment, on the other hand, defines a player's desire to continue participating in sports (Scanlan et al., 1993). These psychological states are considered important sports variables and are frequently examined, as they can facilitate a sports participant's wellbeing and performance (Gillet et al., 2009; Vella et al., 2013; Stenling and Tafvelin, 2014; Kao and Tsai, 2016; Kim and Cruz, 2016; Contreira et al., 2019; Davis et al., 2021).

Overall, the results of these studies provide empirical evidence in understanding sport leadership following the transformational leadership approach as well as identifying how each construct of transformational leadership can influence the sport-related psychological outcomes of Korean players, particularly satisfaction and commitment. Being explicitly aware of this knowledge therefore is vital for sports coaches and practitioners to better predict coach effectiveness. Furthermore, how players respond to the behaviors displayed by coaches can serve as important information that should be considered when creating educational programs designed to optimize the leadership capabilities of coaches. Hence, recognizing which leadership behavior positively or negatively corresponds to player's psychological outcomes can help coaches create effective and tailored strategies that would develop higher levels of coach-player relationships leading to improve psychological states and even performance of players. However, with the numerous leadership studies conducted following the transformational leadership perspective in a sporting environment and using the MLQ to evaluate the leadership behaviors of coaches in Korea, it is surprising that no study has been conducted to consolidate these findings. Given that players' perceptions of satisfaction and commitment can be influenced by the leadership of coaches, it is therefore worth examining the overall strength of the association between transformational leadership behaviors and satisfaction and commitment and the relative strength of each dimension of transformational leadership on these two psychological outcomes by analyzing pertinent quantitative data using meta-analysis. Furthermore, in a previous meta-analysis that examined the relationship between coach leadership styles and two psychological variables that focused on a single instrument (the LSS), it was found that the effects of coach leadership styles and behaviors were large for women but small for men, suggesting a moderating role of the gender of players on the relationship between leadership behaviors and psychological outcomes (Kim and Cruz, 2016). Given these findings, it is plausible that the impact of transformational leadership behaviors on player satisfaction and commitment may also be moderated by gender of players and thereby warrants exploration.

Therefore, the aim of this study was to systematically review the Korean literature related to transformational leadership and to examine the relationships between transformational leadership and satisfaction and commitment. The following are the research questions of this study: (1) What are the overall effect sizes (ES) of the relationship between transformational leadership and satisfaction and commitment? (2) What are the ES of each construct of transformational leadership on the outcome variables?, and (3) What are the ES of the relationships between transformational leadership and the outcomes variables in male and female players?

## Methodology

This meta-analysis was conducted in accordance with Preferred Reporting Items for Systematic reviews and Meta-Analyses guidelines (Panic et al., 2013). The meta-analysis method has been widely adopted by researchers from a variety of scholarly domains to combine findings obtained from studies on similar subjects (Glass, 1976; Dempfle and Loesgen, 2004; Ustunel et al., 2021). Meta-analysis is generally known as analysis of analyses (Glass, 1976). This method generates a general ES and confidence interval for the cumulative evidence resulting from the combination of two or more studies (Borenstein et al., 2021). Effect size is a statistical index that represents the degree of relation among study variables within the study (Hedges and Pigott, 2004; Borenstein et al., 2021). For this study, the random-effects model was adopted because it was assumed that the results of the selected studies were heterogeneous (Petitti, 2001).

### Inclusion Criteria and Coding Data

**Figure 1:**
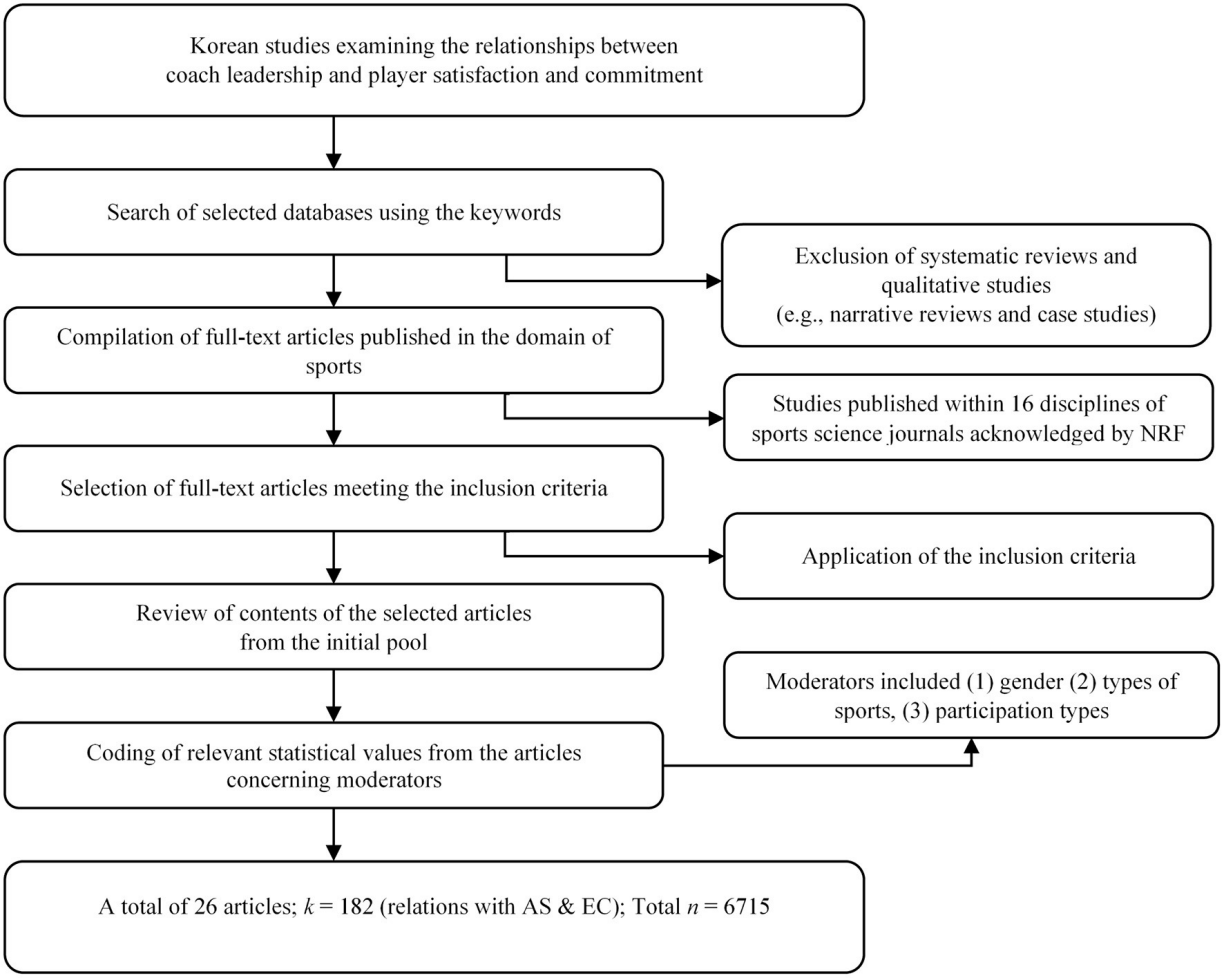
Flow diagram of study selection process.

A systematic search of Korean coaching leadership studies was carried out. To retrieve the literature for this analysis, searches were conducted in Google Scholar and Korean online library databases (i.e., KRpia, DPpia, KISS, and KOSSDA) using the keywords “coach,” “coaching,” “leadership,” “sport(s),” “athlete/athletic satisfaction,” “commitment,” and their Korean equivalents. Once all the references were generated, the titles, keywords, abstracts, and full text versions were screened by the authors. The criteria for inclusion of a study were as follows: (1) full-text articles examining the relationships among study variables (i.e., subdimensions of MLQ, satisfaction, and commitment); (2) studies with adequate sampling sizes and correlation coefficient scores to estimate the standardized ES; (3) studies verifying the gender ratio of sample as moderator. Those that did not contain prevalence data were excluded from the final analyses. At the end of the search and screening, 26 studies were collected from 6,715 samples (Figure 1).

### Data Analysis

In this study, correlation-based ES classification was adopted to calculate the ESs, using Comprehensive Meta-Analysis Ver. 3.0. *I*^2^ statistics were estimated to verify the proportions of inconsistencies across the selected studies that were not explained by random change, and Cochran's *Q* tests were performed to evaluate the heterogeneity among the underlying variables (Supplementary Table 1). For all meta-analyses, the significance level was set at 0.05, upon which the analyses were performed, and as significant heterogeneity was noted across the studies selected, the random-effects model was carried out as suggested by Borenstein et al. (2021). An ES ≥ 0.4 is interpreted as a large ES, 0.25–3.99 a moderate ES, and <0.25 as a small ES (Mayers, 2013).

## Results

The overall ESs of coach leadership behavior, as assessed with the MLQ, for each gender on player satisfaction and exercise commitment are summarized in Table 1.

**Table 1:** Results of meta-analysis of the relationships between coach leadership behavior (MLQ) and player satisfaction and exercise commitment by gender.

	**Gender**	***k***	**ES**	**−95%CI**	**+95%CI**	***Q***	***I*** ^**2**^
Player Satisfaction	Overall	66	0.337	0.277	0.394	330.500	87.800
	Male	40	0.293	0.227	0.356	512.629	92.392
	Female	26	0.380	0.326	0.431	148.388	83.152
Exercise Commitment	Overall	116	0.376	0.333	0.418	415.700	86.900
	Male	86	0.336	0.306	0.367	577.372	85.278
	Female	30	0.415	0.359	0.468	253.941	88.600

*k, number of correlations; Q, the homogeneity statistics; CI, confidence intervals; ES, weighted random effect size*.

**Figure 2:**
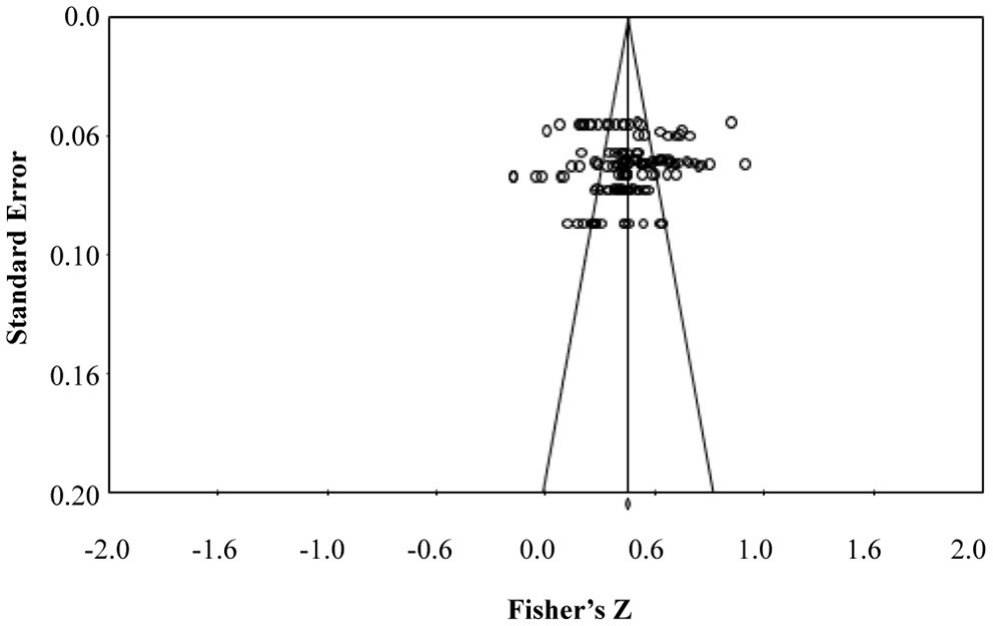
Funnel plot of standard error by Fisher's *Z*.

There were 6,715 participants from 26 studies included in this meta-analysis. The studies selected all used the MLQ as a measurement tool (Bass and Avolio, 1997). The MLQ is the most widely accepted and validated psychologically sound measurement tool for verifying coach leadership behaviors in the field of Korean coaching science. According to the random-effects model, the overall ES of coach leadership behavior on player satisfaction was higher in women (*ES* = 0.380; 95% CIs = 0.326, 0.431; *k* = 26; *p* = 0.05) than in men (*ES* = 0.293; 95% CIs = 0.227, 0.356; *k* = 40; *p* = 0.05). For coach leadership behavior and exercise commitment, it was also found that the ES value of the women (*ES* = 0.415; 95% CIs = 0.359, 0.468; *k* = 30; *p* = 0.05) was higher than that of the men (*ES* = 0.336; 95% CIs = 0.306, 0.367; *k* = 86; *p* = 0.05) (Table 1). *Q* (i.e., an index of variation, “Cochrane's Q”) and *I*^2^ statistics were performed to confirm the homogeneity of the data with a 95% confidence interval (Borenstein et al., 2021). The *Q-statistics* itself cannot be used as the sole index of data heterogeneity because it is only a measure of variation between observed effects and weighted average effects in terms of meta-analysis (Borenstein et al., 2009). According to Higgins et al. (2003), the values of *theI*^2^ statistic (expressed as a percentage with a range from 0 to 100%) are useful and relative criteria for a decision on the degree of heterogeneity. Since most of the *I*^2^ statistics ranged above 75% and all of the significant levels of *Q* statistics were statistically significant, the studies in this meta-analysis were considered to be studies of the same population and deemed to have some degree of heterogeneity, as indicated by Hak et al. (2016). Additionally, we utilized multiple standard methods to examine the data for publication bias and heterogeneity. As depicted in Figure 2, all data points of the selected studies fell within 95% confidence intervals. In other words, we were confident that publication bias was unlikely in our meta-analysis.

Table 2 presents the ESs of the subdimensions of coach leadership behavior on player satisfaction and exercise commitment overall and by gender. The five subdimensions of coach leadership behavior indicated gender differences in the value of ES for both player satisfaction and exercise commitment. The most significant finding from this study was that for players' satisfaction, the sub-dimensions “Charismatic” (*ES* = 0.420; 95% CIs = 0.316, 0.513; *k* = 8; *p* = 0.05), “Individual Consideration” (*ES* = 0.360; 95% CIs = 0.204, 0.498; *k* = 5; *p* = 0.05), “Intellectual Stimulation” (*ES* = 0.421; 95% CIs = 0.348, 0.488; *k* = 8; *p* = 0.05), “Management by Exception” (*ES* = 0.126; 95% CIs = 1.90E−02, 0.265; *k* = 1; *p* = 0.05), and “Contingent Reward” (*ES* = 0.292; 95% CIs = 0.190, 0.387; *k* = 4; *p* = 0.05) showed higher ESs for female players compared with male players. In more detail, the biggest difference in the ES value on player satisfaction between the gender groups was explained by Contingent Reward,” followed by “Intellectual Stimulation,” “Individual Consideration,” “Charismatic,” and lastly “Management by Exception.” The data presented in Table 2 also indicates that the sub-dimension of “Individual Consideration” (*ES* = 0.464; 95% CIs = 0.357, 0.559; *k* = 7; *p* = 0.05) had the highest ES on “Exercise Commitment” for female players followed by “Charismatic” (*ES* = 0.455; 95% CIs = 0.340, 0.557; *k* = 10; *p* = 0.05), “Intellectual Consideration” (*ES* = 0.367; 95% CIs = 0.284, 0.444; *k* = 10; *p* = 0.05), and “Contingent Reward” (*ES* = 0.313; 95% CIs = 0.198, 0.418; *k* = 3; *p* = 0.05).

**Table 2:** Results of meta-analysis of the relationships between subfactors of the MLQ and player satisfaction and exercise commitment by gender.

	**Factor**	**Gender**	***k***	**ES**	**−95%CI**	**+95%CI**	***Q***	***I*** ^**2**^
Player Satisfaction	Charismatic	Overall	26	0.399	0.307	0.484	104.404	87.523
		Men	18	0.378	0.297	0.454	159.940	89.371
		Women	8	0.420	0.316	0.513	48.867	85.675
	Individual Consideration	Overall	13	0.297	0.132	0.447	80.845	91.470
		Men	8	0.234	0.060	0.395	127.056	94.490
		Women	5	0.360	0.204	0.498	34.632	88.450
	Intellectual Stimulation	Overall	18	0.342	0.246	0.432	60.628	81.175
		Men	10	0.263	0.142	0.375	96.544	90.677
		Women	8	0.421	0.348	0.488	24.710	71.672
	Management by Exception	Overall	2	0.121	−0.018	0.254	000	000
		Men	1	0.115	−0.016	0.242	2.46E−15	000
		Women	1	0.126	−0.019	0.265	1.15E−14	000
	Contingent Reward	Overall	7	0.180	0.055	0.301	7.753	67.992
		Men	3	0.068	−0.081	0.214	7.378	72.894
		Women	4	0.292	0.191	0.387	8.127	63.088
Exercise Commitment	Charismatic	Overall	46	0.413	0.328	0.491	212.948	90.457
		Men	36	0.371	0.315	0.423	310.933	88.744
		Women	10	0.455	0.340	0.557	114.963	92.171
	Individual Consideration	Overall	20	0.397	0.295	0.491	93.131	89.385
		Men	13	0.330	0.232	0.422	138.514	91.336
		Women	7	0.464	0.357	0.559	47.747	87.434
	Intellectual Stimulation	Overall	24	0.362	0.280	0.438	83.481	85.736
		Men	14	0.356	0.275	0.432	114.5871	88.654
		Women	10	0.367	0.284	0.444	52.375	82.816
	Management by Exception	Overall	–	–	–	–	–	–
		Men	9	0.277	0.201	0.349	31.085	74.264
		Women	–	–	–	–	–	–
	Contingent Reward	Overall	17	0.312	0.214	0.404	54.981	77.259
		Men	14	0.311	0.228	0.390	103.896	87.487
		Women	3	0.313	0.198	0.418	6.066	67.031

*k, number of correlations; Q, the homogeneity statistics; CI, confidence intervals; ES, weighted random effect size*.

## Discussion

This study examined the influence of coach transformational leadership, as assessed by the MLQ instrument, on player satisfaction and commitment using a systematic meta-analysis approach. The overall results showed that transformational leadership had significant positive and moderate effects on both player satisfaction and commitment. This finding indicates that player satisfaction and commitment can be moderately enhanced when coaches frequently demonstrate transformational leadership behaviors such as actively listening to players' needs and concerns, articulating the vision of the team in an inspiring way, challenging players to go beyond their capacities, and showing admirable behaviors. This result is consistent with previous studies that also found a positive relationship between transformational leadership and members' psychological outcomes of satisfaction and commitment (Chin, 2007; Jackson et al., 2013; Nohe and Hertel, 2017; Gui et al., 2020).

### Transformational Leadership Constructs

Among the constructs of transformational leadership, charismatic and intellectual stimulation yielded positive and moderate effects on player satisfaction. Also, Individual consideration, contingent reward, and management by exemption were positively correlated with satisfaction, but with small ESs. This result suggests that all leadership behaviors, whether transformational or transactional, can positively affect player satisfaction. The present findings corroborate previous meta-analyses conducted by Chin (2007); Nohe and Hertel (2017), and Gui et al. (2020), who found a positive association between transformational leadership and satisfaction. However, previous investigations have not addressed how each leadership construct contributes to followers' satisfaction levels. The current study also supports the results of a meta-analysis on the relationship between coach leadership and satisfaction (Kim and Cruz, 2016). However, the data analyzed came from articles that used the LSS as a measurement tool to assess leadership behaviors. Hence, our study results not only go beyond the direct effect between transformational leadership and satisfaction, but also provide a different perspective in understanding leadership in sports by examining the relative strength of the influence of leadership behaviors on player athletic satisfaction based on the constructs assessed by the MLQ.

Similarly, all five constructs of leadership had a significant and positive relationship with player commitment. Interestingly, the strength of the relationship between each leadership construct and commitment yielded moderate-to-large ESs. This finding suggests that when coaches generally inspire, motivate, intellectually stimulate, provide positive feedback and reinforcement, and influence players to achieve their sports goals, the higher the desire of players to continue their sports participation or stay committed with the sport/team. Therefore, coaches should emphasize these behaviors to ensure that players maintain high level of sport commitment. This result confirms similar studies that indicated that the charismatic stimulation, intellectual stimulation, individual consideration, contingent reward, and management by exemption constructs of leadership positively influence the commitment level of followers (Jackson et al., 2013).

A noteworthy finding of this study was the stronger effects of transformational leadership behaviors on player commitment compared with satisfaction. In particular, management by exception and contingent reward had moderate effects on commitment, whereas they only had small effects on satisfaction. These findings indicate that players tend to show higher commitment in their athletic participation rather than feel satisfied with their sports experiences when coaches display more frequent transactional behaviors in addition to transformational leadership behaviors. This result may be attributed to the player's individual traits, particularly their sensitivity to environmental and motivational stimuli that can facilitate the enhancement of sport commitment. When coaches instruct and give feedback to players based on their athletic needs and appropriately reward performance accomplishments, players who are receptive to these external and motivational stimuli would clearly notice the positive behavioral cues exhibited by their coaches. Consequently, these players are more likely to have a higher desire to stay committed and continue sports participation since the transformational leadership behaviors that coaches display are more congruent with their needs and motivations. This notion is in line with those of other scholars who proposed that one's sensitivity toward environmental stimuli, in this case, the degree of perceptions toward the leader's individual consideration and contingent reward leadership, may be influenced by a person's characteristics (De Meyer et al., 2016; Stenling et al., 2017). Hence, aside from the demonstrating transformational leadership behaviors, coaches are advocated to provide higher degree of instructional feedback and to appropriately reward performance accomplishments of players, since players who are receptive to these external and motivational stimuli would clearly notice the positive behavioral cues exhibited by their coaches and eventually have higher desire to continue sports participation.

Another plausible reason for players' higher level of perceived commitment than satisfaction is due to coach transformational behaviors that are aligned to players' preference. Studies showed that elite level and mature players favor a coach that is efficient, well-structured, encouraging, and constructive in giving feedback (Weinberg and Gould, 2015; Cruz and Kim, 2017), which relatively depict behaviors relating to active management by exception and contingent reward. Since players included in the selected studies were mostly competitive Korean players, it seemed that their perceived commitment level were greatly affected when coaches showed not only idealized influence, intellectual stimulation, inspirational motivation, and individual consideration behaviors, but more so when coaches displayed constant management by exception and contingent reward leadership behaviors. Based on the current results, for players to stay highly positive and committed to their sport participation, coaches should not only inspire and challenge players to accomplish their goals, but also identify players' strengths and weaknesses in order to create training programs that are aligned with their needs that would further optimize their performance. The current finding also verifies the concept of transformational leadership that personal motivation and morale of followers are enhanced when leader offer tasks to followers that would further develop their strengths as well improve their limitations (Bass, 1997).

### Gender Differences in the Relationships Between Transformational Leadership Behaviors and Player Satisfaction and Commitment

The results showed that the transformational leadership of coaches has significant and positive effects on satisfaction and commitment of players but with greater effect on women than men. Moreover, all the leadership constructs had larger ES in women compared with men, particularly intellectual stimulation and charismatic leadership for satisfaction and individual consideration and charismatic leadership for commitment. This result suggests that female players' athletic satisfaction and commitment are likely to increase to a greater extent than male players when they perceive that their coaches frequently demonstrate leadership behaviors that are charismatic, intellectually stimulating, considerate, and kind with regard to their sport participation and performance. The difference in the magnitudes of the relationships between leadership behaviors, and satisfaction and commitment in male and female players may be explained by variations in their preferences for these leadership behaviors and how they correspond (or diverge) with their actual perceptions of coach behaviors. In a study on leadership preferences in collegiate athletes, men reported a higher preference for social support and autocratic behavior, whereas women conveyed a stronger preference for positive feedback and situational consideration (Witte, 2011). Koh and Wang (2015) reported that the behaviors of coaches related to goal setting, mental preparation, and competition strategies were greatly perceived by male players compared with female players. These specific behaviors were also found to be positively correlated with satisfaction. Rodrigues et al. (2020) found that coaches who emphasized task and ego motivational climates positively facilitated the psychological satisfaction and behavioral regulation of male players, but not female players. Hence, male players who preferred more autocratic or transactional leadership and a lower degree of transformational leadership behaviors but perceived their coaches as demonstrating too much or too little of these preferred behaviors tended to be less satisfied and committed with their athletic participation because the actual behaviors of the coaches did not match their preferences and vice versa. In contrast, female players in the present study could have had coaches who demonstrated positive leadership behaviors, such as intellectual stimulation, charismatic stimulation, and individual consideration, which were congruent with their preferences, resulting in higher commitment and satisfaction. This notion is supported by a previous meta-analysis (Kim and Cruz, 2016) that showed that the magnitude of the relationship between coach leadership and athletic satisfaction was stronger in women than in men. In particular, females perceived higher levels of satisfaction when their coaches exhibit a lower degree of autocratic behavior but higher degrees of positive feedback, social support, and training and instruction.

Another possible reason for the stronger influence of coach leadership behaviors on female player satisfaction and commitment compared with males is coach-player gender interaction. Cruz and Kim (2017) demonstrated that female players with male coaches tended to prefer democratic, autocratic, and social support leadership behaviors more than those with female coaches. Murray et al. (2018) found that male coaches supervising female players were perceived to display a greater level of relationship quality (complementarity). They also reported that female players perceived their coaches, whether male or female, as displaying a greater level of affective empathy. Hence, the gender difference in the degree of perceived satisfaction and commitment in players may have been brought about by coaches who emphasized and promoted greater interpersonal relationships when interacting with players *via* their leadership behaviors. These interpersonal relationship-promoting behaviors were then clearly observed and positively perceived by female players than by males because these leadership behaviors were generally preferred by females.

Overall, the present findings underscore the importance of congruency between player preferred and perceived actual leadership behaviors as well as the coach-player dyad, particularly opposite gender interaction (e.g., male coach-female player), in developing positive psychological states in sports players. The current results also support both the Multidimensional Model of Leadership (Chelladurai, 1990) and transformational leadership perspectives which posit that positive changes are likely to occur when leader behaviors are congruent with followers/members preferences and when leaders align responsibilities to followers based on their strengths and weaknesses (Bass, 1990; Newland et al., 2015).

### Theoretical and Practical Implications

The results of this study contribute to the sport psychology and sports leadership literature by shedding light on the overall direct effect of coach leadership behavior on player satisfaction and commitment by consolidating previous studies and examining them using meta-analysis. In other words, transformational leadership can positively enhance players' psychological states. Recently, coach leadership has been identified as an important factor in creating a sustainable sports environment. A sustainable sports environment is described as a sport setting wherein coaches provide players with well-planned training programs based on skill level, support players' personal development, build sincere relationships with players, and focus more on their wellbeing and health rather than performance results (Dohsten et al., 2020; Dohlsten et al., 2021). Therefore, in order to create a more sustainable environment for players, coaches and sport practitioners working with players should be aware of and frequently demonstrate transformational leadership behaviors that inspire and motivate players to achieve their sport-related objectives and encourage players to accomplish these target goals beyond expectations. In such manner, players would not feel disappointed or resentful, or experience a sense of futility with their sports experiences (i.e., not accomplishing their goals), but rather feel proud and gratified with their accomplishments, view failures as opportunities for growth, and continue with their sports participation. Furthermore, as coaches are also found to cause dropout among players in competitive and elite sports (Andronikos et al., 2019; Thomas et al., 2021), the leadership role of the coach in providing support and motivation to the overall wellbeing of players is even more vital in order to sustain or further enhance players' commitment and satisfaction level.

Using a meta-analysis approach, this study sheds new light on identifying the contribution of each transformational leadership construct measured by MLQ in influencing player satisfaction and commitment, thereby extending current knowledge in sport leadership in general and transformational leadership in particular. It is therefore suggested that coaches who want their players to have a high level of satisfaction and commitment or strive to transform players' sports-related attitudes, values, and morale should display a high degree of charismatic, intellectual stimulation, and individual consideration leadership behaviors for these behaviors are found to have large to moderate effects in developing positive psychological states in players.

Finally, by examining player gender as a moderating factor in the relationship between transformational leadership and satisfaction and commitment, the present study provides additional knowledge on how the overall influence and each construct of coach transformational leadership on player levels of satisfaction and commitment may vary between male and female players. The findings showed that the satisfaction and commitment of female players are positively affected to a greater extent than those of male players when coaches display transformational leadership behaviors. As such, coaches should be mindful of how to appropriately interact with male and female players by showing more frequent individual consideration, charismatic, and intellectual stimulation leadership behaviors to female players because they are more sensitive to these behaviors, resulting in higher levels of satisfaction and commitment than males. To achieve this in a Korean sport setting for instance, coaches should neither ignore nor insult ideas and opinions when handling female players. Rather, they should promote open communication, provide positive guidance and encouragement, and show trust and respect. On the other hand, coaches working with male players may also provide players with previous suggestions, but at the same time focus more on giving direct instructions, constructive feedback, and contingent rewards. Finally, a formal and regular leadership development program is a viable approach to help coaches optimize their attitude- and relationship-building competencies, so they know what effective strategies are appropriate to use when interacting with players with distinct qualities and needs, how to employ these strategies, and when to implement these plans that would facilitate positive sport-related outcomes and avoid turnover.

### Limitations and Future Directions

While the study provided new perspectives on leadership in sports, there are also some important limitations. First, since the study only consolidated previous studies that used a multifactor leadership questionnaire instrument to evaluate the transformational leadership of coaches, the results are limited to the constructs within this measurement tool. Hence, it is suggested that future studies should examine other instruments that also assess transformational leadership in sports, such as the Transformational Leadership Inventory (TLI) (Podsakoff et al., 1990) and determine not only the overall but also each construct's impact on the psychological states of players.

Second, the published articles all came from Korean journal publications, and thus the findings can only be generalized to this set of population and sport settings. However, the results may also be applicable to countries with similar cultures, particularly those with high collectivism. As such, a cross-cultural approach in examining the relationships between transformational leadership and both player satisfaction and commitment may be a worthy endeavor. A previous meta-analysis (Jackson et al., 2013) has shown that culture is a significant moderator between transformational leadership and the psychological state of followers.

Lastly, the present meta-analysis was limited to the influence of transformational leadership on the psychological outcomes of player athletic participation. Other outcomes related to transformational leadership, such as cohesion (Cronin et al., 2015; Bosselut et al., 2018; Erikstad et al., 2021) and wellbeing (Stenling and Tafvelin, 2014; Bormann et al., 2016; Krukowska, 2016) were not examined because of the lack of published articles that used the MLQ as the main instrument. Hence, future meta-analytic research will be possible if there are sufficient numbers of articles to conduct such examinations.

## Conclusions

This meta-analysis found that transformational leadership has a positive and moderate impact on athletic satisfaction and commitment. Transformational leadership constructs, particularly charismatic and intellectual stimulation, have moderate effects, whereas individual consideration, contingent reward, and management by exemption have small effects on player satisfaction. Charismatic behavior has a large impact, whereas individual consideration, intellectual stimulation, contingent reward, and management by exemption have moderate effects on player commitment. Finally, the positive impact of transformational leadership behaviors on player satisfaction and commitment was stronger in female players than in male players. Hence, effective leadership in sports is dependent on the interaction among leadership behaviors of the coach, personal characteristics of the players, and situational factors and highlights the importance of transformational leadership as an important requirement for creating a more positive and sustainable sports environment.

## Data Availability Statement

The original contributions presented in the study are included in the article/supplementary material, further inquiries can be directed to the corresponding author/s.

## Author Contributions

HK and AC conceptualized the research project and contributed to the writing of the manuscript (from the initial draft to the final manuscript). HK analyzed the data. Both authors contributed to the article and approved the submitted version.

## Funding

This research was supported by the Bisa Research Grant of Keimyung University in 2021.

## Conflict of Interest

The authors declare that the research was conducted in the absence of any commercial or financial relationships that could be construed as a potential conflict of interest.

## Publisher's Note

All claims expressed in this article are solely those of the authors and do not necessarily represent those of their affiliated organizations, or those of the publisher, the editors and the reviewers. Any product that may be evaluated in this article, or claim that may be made by its manufacturer, is not guaranteed or endorsed by the publisher.
