# Supplementary material for: Gender Differences in the Relationships Between Coach Transformational Leadership and Player Satisfaction and Commitment: A Meta-Analytic Review
Source: Front Psychol. 2022 Jun 21;13:915391. doi: 10.3389/fpsyg.2022.915391 (PMC9253673; doi:10.3389/fpsyg.2022.915391)
Supplement: Supplementary file 1 [file Table_1.DOCX]

Supplementary Table. List of studies included in the meta-analysis

| Authors | Year | Outcome |
| --- | --- | --- |
| Bum et al. | 2015 | Commitment |
| Choi and Song | 2015 | Commitment and Satisfaction |
| Choi and Kim | 2011 | Satisfaction |
| Cho and Sung | 2009 | Commitment and Satisfaction |
| Choi et al. | 2009 | Commitment |
| Choi at al. | 2009 | Satisfaction |
| Eun, H.G. | 2009 | Satisfaction |
| Hur, S.E. | 2010 | Satisfaction |
| Jang et al. | 2011 | Commitment and Satisfaction |
| Jeon et al. | 2009 | Commitment |
| Kim, M.H. | 2012 | Commitment and Satisfaction |
| Kim, Y.K. | 2014 | Commitment |
| Kim and Won | 2012 | Commitment |
| Kim et al. | 2010 | Commitment |
| Kim et al. | 2018 | Satisfaction |
| Lee and Yeo | 2013 | Commitment and Satisfaction |
| Lee et al. | 2011 | Commitment |
| Na and Lee | 2011 | Satisfaction |
| Park and Jung | 2020 | Commitment |
| Roh et al. | 2017 | Commitment |
| Ryu and Park | 2021 | Commitment |
| Seo, J.H. | 2010 | Commitment |
| Song et al. | 2014 | Commitment |
| Sun and Lee | 2019 | Commitment and Satisfaction |
| Yoo and Hwang | 2017 | Commitment |
| Yu and Park | 2011 | Commitment |
